# Comparative Evaluation of Peripheral Blood Neutrophil to Lymphocyte Ratio, Serum Albumin to Globulin Ratio and Serum C-Reactive Protein to Albumin Ratio in Dogs with Inflammatory Protein-Losing Enteropathy and Healthy Dogs

**DOI:** 10.3390/ani13030484

**Published:** 2023-01-30

**Authors:** Federica Cagnasso, Antonio Borrelli, Enrico Bottero, Elena Benvenuti, Riccardo Ferriani, Veronica Marchetti, Piero Ruggiero, Barbara Bruno, Cristiana Maurella, Paola Gianella

**Affiliations:** 1 Department of Veterinary Sciences, University of Turin, Largo P. Braccini 2, 10095 Grugliasco, Italy; 2 Endovet Italia, Via A. Oroboni, 00100 Rome, Italy; 3 Department of Veterinary Sciences, University of Pisa, Via Livornese Lato Monte, 56121 Pisa, Italy; 4 Istituto Zooprofilattico Sperimentale del Piemonte, Liguria e Valle d’Aosta, Via Bologna, 220, 10154 Torino, Italy

**Keywords:** dog, chronic enteropathy, inflammatory bowel disease, leukocytes, clinical response

## Abstract

**Simple Summary:**

Canine inflammatory protein-losing enteropathy caused by immunosuppressive-responsive enteropathy (IRE-PLE) is associated with a time-consuming diagnostic workup and a guarded prognosis. In human medicine, neutrophil to lymphocyte ratio, albumin to globulin ratio and C-reactive protein to albumin ratio are routinely available biomarkers that have been shown to correlate with several clinical parameters and a poor prognosis. Scattered information exists on the use of these three biomarkers in dogs with IRE-PLE. This study evaluated the clinical significance of these biomarkers in a population of dogs with IRE-PLE at the time of diagnosis and after therapy. Increased values of all three biomarkers were detected in dogs with IRE-PLE at the time of diagnosis, and correlations were observed between some of these biomarkers and the existing chronic enteropathy activity index. After therapy, changes in all three biomarkers were observed. Further studies are needed to assess their clinical significance at a longer follow-up.

**Abstract:**

Few routinely available biomarkers are clinically useful in assessing dogs with inflammatory protein-losing enteropathy caused by immunosuppressive-responsive enteropathy (IRE-PLE). Only the neutrophil to lymphocyte ratio (NLR) has been studied, while no information exists on the use of the albumin to globulin ratio (AGR) and C-reactive protein to albumin ratio (CRP/ALB). We aimed to evaluate the clinical significance of the NLR, AGR and CRP/ALB in a population of dogs with IRE-PLE. The medical records of 53 IRE-PLE dogs were reviewed at the time of diagnosis (T0) and 1 month after the initiation of immunosuppressants (T1). A control group of 68 healthy dogs was used for comparison. At T0, the median values of the NLR and AGR of sick dogs were significantly higher and lower than those of healthy dogs, respectively. With the increase in the chronic enteropathy activity index, AGR and CRP/ALB significantly decreased and increased, respectively. At T1, NLR and AGR significantly increased, while CRP/ALB significantly decreased. NLR, AGR and CRP/ALB did not differ significantly between dogs classified as responders and nonresponders according to the chronic enteropathy activity index. Further studies are needed to provide more information on this subject.

## 1. Introduction

Protein-losing enteropathy (PLE) is a syndrome characterized by the abnormal loss of serum proteins into the gastrointestinal lumen. Any gastrointestinal disease, if severe enough, can lead to intestinal protein loss [1,2,3,4]. Isolated albumin loss can be observed, while hypoproteinemia associated with the loss of albumin and globulin is the most common clinicopathological abnormality. The present study focuses on a population of dogs with inflammatory PLE caused by immunosuppressant-responsive enteropathy (IRE) [5,6]. Diagnosis of IRE is currently defined by chronic persistent or recurrent gastrointestinal signs; histopathologic evidence of mucosal inflammation; exclusion of other causes of gastrointestinal inflammation; inadequate response to dietary, antibiotic and anthelmintic therapies alone; and clinical response to steroids and/or other immunosuppressants [4,5]. Some dogs show no adequate response to immunosuppressants, so the enteropathy is categorized as nonresponsive (NRE) [5]. Therefore, since the diagnostic workup is time-consuming and therapy has still not been standardized, tailored and easily accessible biomarkers that aid in the diagnosis, monitoring of disease activity and prognostication could be helpful particularly if owners’ finances are limited.

Inflammatory cells (e.g., neutrophils and lymphocytes) and plasma proteins are involved in the regulation of the immune response, inflammation, protection against infection and repair and recovery of damaged tissue [7,8,9]. In addition, some proteins function to bind drugs with important implications in clinical therapy [10]. Therefore, the impaired cellular function and plasma protein variation during inflammation may result in limited bacterial clearance, fuel an ongoing inflammatory response and influence the clinical outcome. Ratios between different circulating immune cells (neutrophil to lymphocyte ratio (NLR), monocyte to lymphocyte ratio (MLR) and platelet to lymphocyte ratio (PLR)) have been studied in human medicine as prognostic markers in several disorders [11,12]. In particular, it has been observed that the NLR increases in patients with active forms of IBD [13,14,15,16]. The NLR also appears to be correlated with several clinical parameters [14,17]. In the veterinary counterpart, only a few studies have explored the diagnostic value of NLR in canine CE, showing a positive correlation with disease activity [18,19].

Recently, the serum albumin to globulin ratio (AGR) has been used as a biomarker of poor prognosis in both inflammatory and neoplastic diseases [20,21,22]. The serum levels of albumin, which is a negative acute-phase protein, decrease following inflammation and poor nutritional status, whereas those of globulin increase. As a consequence, the AGR decreases [23,24]. In the veterinary counterpart, the AGR has proven useful in aiding the diagnosis of some neoplastic and inflammatory conditions [25,26,27].

C-reactive protein (CRP) is a positive acute-phase protein, which serves as a nonspecific marker of inflammation and disease severity both within the veterinary and human medical fields [28,29,30,31]. In human medicine, the C-reactive protein-albumin ratio (CRP/ALB) has been used in emergency medicine, oncology and gastroenterology, with a high discriminative capacity for active inflammatory bowel disease (IBD) [32,33,34,35]. In the veterinary counterpart, the plasma and serum CRP concentrations have been found to be significantly higher in dogs with IBD compared with those in healthy control dogs and higher in dogs with inflammatory PLE than in those with food-responsive diarrhea [36,37]. Moreover, increased serum CRP concentration seems to be associated with an increased risk of death or euthanasia in dogs with PLE [36,37]. However, to the best of the authors’ knowledge, no information on the use of NLR, AGR and CRP/ALB in dogs with CE and inflammatory PLE exists.

Based on these premises, further investigations are necessary in veterinary gastroenterology. Therefore, the aim of this retrospective study was to evaluate the possible clinical significance of the NLR, AGR and CRP/ALB in a population of dogs with inflammatory PLE.

## 2. Materials and Methods

The medical records of 53 dogs with inflammatory PLE secondary to IRE (IRE-PLE) diagnosed at the Veterinary Teaching Hospital of Turin University, Italy, between January 2019 and January 2022 were retrospectively reviewed. The owners were informed about the use of clinical data for scientific purposes and gave their consent.

In total, 26 dogs (49.1%) were female (13 spayed), and 27 dogs (50.9%) were male (3 neutered). Twenty-two different breeds were included: German Shepherd (n = 9), Belgian Malinois (n = 3), Jack Russel Terrier (n = 3), Yorkshire Terrier (n = 3), American Staffordshire terrier (n = 2), Australian Shepherd (n = 2), Border Collie (n = 2), English Setter (n = 2), Golden Retriever (n = 2), Maltese (n = 2), Pug (n = 2), American Pit Bull Terrier (n = 1), Bernese Mountain Dog (n = 1), Cavalier King Charles Spaniel (n = 1), Chihuahua (n = 1), Cocker Spaniel (n = 1), Dachshund (n = 1), Dobermann Pinscher (n = 1), Dogo Argentino (n = 1), Greyhound (n = 1), Italian Mastiff (n = 1), Labrador Retriever (n = 1) and Podenco (n = 1). Nine dogs were mixed-breed. The median age was 84 months (68–108).

Inclusion criteria were historical and physical examination findings, chronic gastrointestinal signs lasting for more than 3 weeks, hypoalbuminemia of gastrointestinal origin (≤2.8 g/dL) and histopathological evidence of gastrointestinal inflammation on multiple biopsies collected by endoscopy. The histologic examination must have been performed according to the histopathological standards of the World Small Animal Veterinary Association (WSAVA) Gastrointestinal Standardization Group [4]. Fecal flotation and Giardia antigen test, complete blood count, serum biochemistry, pre- and postprandial bile acids, serum basal cortisol or ACTH stimulation test, trypsin-like immunoreactivity, pancreas-specific lipase levels, serum folate and cobalamin concentrations, urinalysis including the urinary protein to creatinine ratio and ultrasound examination were required to rule out infectious, parasitic, liver and pancreatic diseases, along with intestinal diseases of other etiology and extraintestinal diseases. Dogs responding to dietary trials with selected or hydrolyzed protein diets for at least 2 weeks were excluded. Dogs responding to microbial manipulation treatment strategies (i.e., pre-, pro-, syn- and postbiotics, fecal microbiota transplantation and antibiotics) were excluded from the analysis. Furthermore, dogs were excluded if they had received anti-inflammatory/immunosuppressive treatments and/or antibiotics within 4 weeks before the endoscopic procedure (T0). Information gleaned from the medical records at T0 on the chronic canine enteropathy clinical activity index (CCECAI) score [3], body weight (kg), complete blood count (ADVIA 120 Siemens, Italy), serum concentrations of total protein and albumin (BT 3500 VET Futurlab, Italy) and serum C-reactive protein (CRP) concentration was studied. The CCECAI scores were taken from the medical records; however, when this was not found, a CCECAI score was calculated retrospectively using the serum albumin concentration, presence or absence of peripheral edema and peritoneal effusion on ultrasound examination and the owner’s scores on appetite, activity level, vomiting, fecal consistency and frequency, weight loss and pruritus. When the information was not available to calculate CCECAI, the score was not given. Four disease severity groups were identified based on the CCECAI scores: mild disease (CCECAI 4–5; MI), moderate disease (CCECAI 6–8; MO), severe disease (CCECAI 9–11; S) and very severe disease (CCECAI ≥ 12; V) [3]. Information from complete blood count was considered only if confirmed by manual cell differential count by a clinical pathologist. Hypoalbuminemia was further classified as mild (1.5 to 2.8 g/dL; MIA), moderate (1.2 to 1.49 g/dL; MOA) and severe (<1.2 g/dL; SA). The NLR was calculated as the ratio between absolute neutrophils and lymphocytes. The CRP/ALB was determined by dividing the serum CRP concentration by the serum albumin concentration. The serum globulin concentration was calculated as the difference between the serum total protein concentration and the serum albumin concentration. The AGR was determined by dividing the serum albumin concentration by the serum globulin concentration.

The immunosuppressive therapy was based on oral prednisolone (1 mg/kg q12h for 2–3 weeks before considering dose reduction). In some dogs, it was associated with oral chlorambucil (2–6 mg/m^2^ q24h for at least 2 weeks before considering dose reduction) at the discretion of the clinician. Selected protein or hydrolyzed diets were given to all dogs, in addition to multistrain probiotics. Parenteral cobalamin and oral folate supplementations were based on their serum concentrations. Dogs that required modification of the type of immunosuppressive drug, diet and supplementations between T0 and T1 were excluded.

Follow-up information was obtained from the medical records 1 month after the start of immunosuppressants (T1). The blood samples at T0 had to be drawn the day of the endoscopic procedure or within 10 days prior, and the blood samples at T1 had to be drawn 1 month after the start of immunosuppressants. The CCECAI scores at T1 were used to classify dogs into responders and nonresponders. Dogs were classified as responders in case of a reduction in CCECAI scores > 25%, whereas they were classified as nonresponders in case of a reduction in CCECAI scores < 25% or death [38]. Dogs that died due to IRE-PLE-unrelated causes or dogs for which CCECAI scores were not available at T1 were removed from statistical analyses.

Information from 68 healthy owned dogs presented at the same Veterinary Teaching Hospital of the study group for their annual check-up and vaccination or preanesthetic evaluation before neutering served as a retrospective control group. Healthy dogs were enrolled based on unremarkable history and physical examination, no ongoing drug administration, normal complete blood count and serum biochemistry. In total, 39 dogs of different breeds and 29 mixed-breed dogs were included. Of those, 25 dogs were female (13 spayed), and 43 dogs were male (11 neutered). The median age was 72 months (24–108).

### Statistical Analysis

Data were analyzed for the assumption of normality using a test based on skewness and another test based on kurtosis. Continuous and nonparametric variables are presented as medians and interquartile ranges (IQRs); categorical data are reported as counts and percentages. A Wilcoxon rank-sum test and a Kruskal–Wallis test were performed to compare 2 or ≥3 groups (unpaired data). The equality of matched pairs of observations at time T0 versus time T1 was evaluated by using the Wilcoxon matched-pairs test. The association between continuous variables and the NLR was tested using the Spearman correlation coefficient ρ. To study the relationship between selected clinicopathological variables (NLR, albumin, CRP, CRP/ALB and AGR) and the CCECAI scores, a robust regression analysis was performed. The same analysis was performed to study the relationship between NLR and selected clinicopathological variables (albumin, CRP, CRP/ALB and AGR) and to study the relationship between NLR and individual features (sex and age) in both control and study groups. Receiver operating characteristic (ROC) curves were obtained; the sensitivity and specificity of the NLR, CRP/ALB and AGR were calculated using the likelihood ratio in order to distinguish responder and nonresponder dogs. The *p*-value was set at 0.05. The statistical analysis was performed using a statistical software package (Stata SE 17.0).

## 3. Results

### 3.1. Selected Clinicopathological Characteristics

Table 1 and Table 2 show selected clinicopathological characteristics of healthy dogs and dogs with IRE-PLE, respectively. At T0, the median NLR of dogs with IRE-PLE was significantly higher than the median NLR of healthy dogs (*p =* 0.00). The median AGR of dogs with IRE-PLE was significantly lower than the median AGR of healthy dogs (*p =* 0.006).

### 3.2. Association of the CCECAI Score with NLR, Albumin, AGR and CRP/ALB at T0

**Figure 1:**
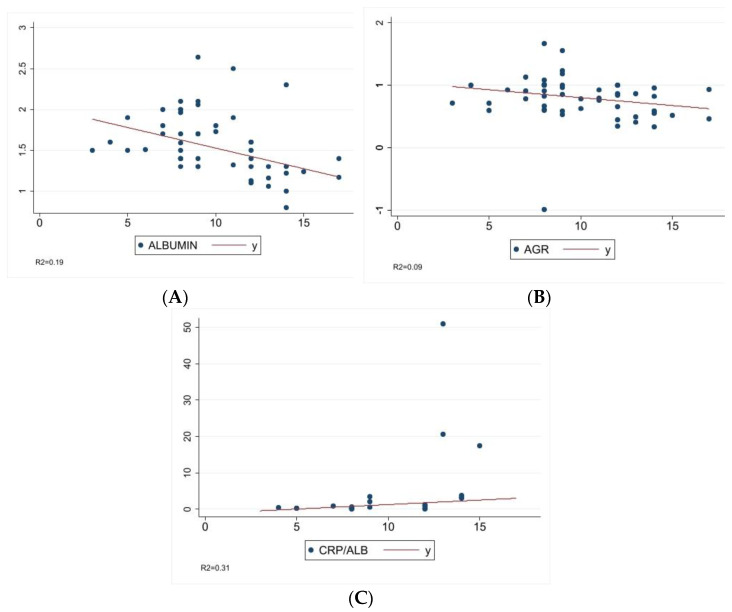
The relationship between the CCECAI score and the serum concentrations of albumin (**A**), AGR (**B**) and CRP/ALB (**C**) before endoscopic procedure (T0) are graphically represented. The single parameter is on the *Y* axis, whereas the CCECAI score lies on the *X* axis.

The study of the relationship between the CCECAI score and serum concentrations of albumin, AGR and CRP/ALB resulted in significative associations (Table 3). The serum concentrations of albumin and AGR significantly decreased with the increase in the CCECAI score (Figure 1A,B), while the CRP/ALB significantly increased (Figure 1C).

**Figure 2:**
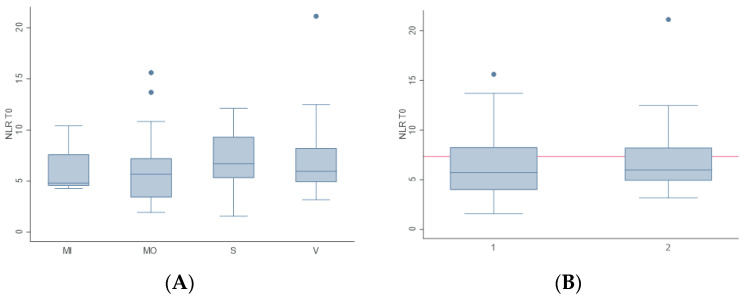
The relationship between the disease severity groups (MI, MO, S and V) and the NLR are graphically represented (**A**). The red line represents the optimal NLR cutoff to distinguish the group with very severe clinical signs (V = 2) from the other groups (MI + MO + S = 1) as given by the nonparametric ROC analysis (**B**).

No correlations were found between the median CCECAI score and NLR (*p* = 0.5) and between the four disease severity groups (MI, MO, S and V) and NLR (*p* = 0.8) (Figure 2A). No significant differences were found even by distinguishing the group with very severe clinical signs (V = 2) from the other groups (MI + MO + S = 1) (Figure 2B).

### 3.3. Association of the NLR with Albumin, AGR and CRP/ALB at T0

**Figure 3:**
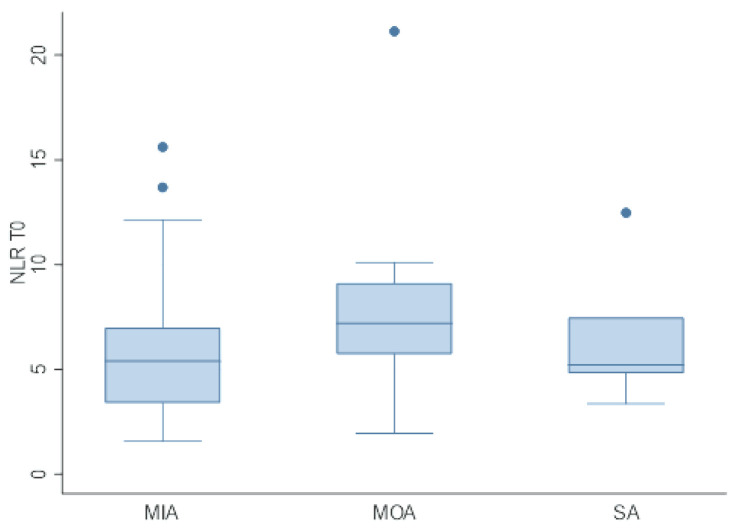
Distribution of NLR within the hypoalbuminemia classes at T0. The NLR is on the *Y* axis, whereas the hypoalbuminemia classes (MIA = mild hypoalbuminemia, MOA = moderate hypoalbuminemia and SA = severe hypoalbuminemia) are on the *X* axis.

Thirty-two dogs had a normal neutrophil count (60.4%) and forty-seven had a normal lymphocyte count (88.7%). The neutrophil and lymphocyte counts were normal in 26 (49%) dogs. Neutrophilia and lymphopenia were detected in 20 (37.7%) and 6 (11.3%) dogs, respectively. Mild neutropenia was seen in 1 dog (1.9%). Lymphocytosis was not observed. The study of the association of NLR with the serum concentrations of albumin, AGR and CRP/ALB resulted in a mild negative correlation with the median serum albumin concentration (*p* = 0.04). The performance of the Kruskal–Wallis test did not evidence any significative difference among the NLR and the three hypoalbuminemia classes (MIA, MOA and SA) (*p* = 0.09) (Figure 3). No correlations were found between NLR, AGR and CRP/ALB.

### 3.4. Comparison of the CCECAI Score, NLR, Albumin, AGR and CRP/ALB before Endoscopic Procedure (T0) and 1 Month after the Initiation of Immunosuppressants (T1)

One month after the initiation of immunosuppressants (T1), 28 dogs were classified as responders and 23 dogs as nonresponders. For the remaining two dogs, the CCECAI scores were not available. The median CCECAI score was 5 (range 3–8), the median NLR was 6.7 (range 3.9–12.2), the median serum concentration of albumin was 2.1 g/dL (range 1.5–2.62), the median AGR was 0.93 (range 0.79–1.075) and the median CRP/ALB was 0.3 (range 0.04–0.87). Results of the comparison of the CCECAI score, NLR, serum concentration of albumin, AGR and CRP/ALB before the endoscopic procedure (T0) and 1 month after the initiation of immunosuppressants (T1) are shown in Table 4. NLR, serum concentrations of albumin and AGR were significantly increased, while the CCECAI score and CRP/ALB were significantly decreased. NRL, AGR and CRP/ALB were not statistically different between responders and nonresponders.

## 4. Discussion

This retrospective study aimed to explore the clinical significance of the NLR, AGR and CRP/ALB in a population of dogs with IRE-PLE.

In dogs with CE, scattered information on the role of NLR exists and, to the best of the authors’ knowledge, studies that focus exclusively on inflammatory PLE are lacking. Based on previous observations, IRE dogs show higher NLR compared to healthy dogs, as well as nonresponders compared to responders [18,19]. Moreover, an NLR ≥ 5.5 can potentially aid in distinguishing dogs with food-responsive enteropathy from dogs with IRE. However, no difference in NLR has been detected between dogs with IRE and dogs with nonresponsive enteropathy [19]. In the present study, dogs with IRE-PLE showed higher NLR compared to healthy dogs and dogs from two previous studies. [18,19]. The slightly higher NLR values observed here might be explained by having included only dogs with PLE, generally causing severe inflammation of the intestinal tract. Despite IRE being a diagnosis by exclusion, an effect of comorbidity on increased NLR values, such as pancreatitis, as well as the effect of stress associated with the hospital visit, cannot be excluded in all of the dogs from this retrospective study [39]. Lymphopenia is also a common finding in dogs with PLE, and this could further have contributed to an increase in the NLR [40]. Although a moderate positive correlation between the NLR and the CCECAI score at diagnosis was found before [18,19], no significant correlations were found in this study either with the median CCECAI score or with the disease severity groups. These results might be explained by the low sensitivity and specificity rates of the NLR, which is not as effective as other biomarkers for determining active IBD [15,41]. Moreover, considering that most of the dogs of this study showed elevated CCECAI for which high NLR values would have been expected, the lack of correlation between the NLR and the CCECAI score further questions the clinical utility of the NLR in IRE-PLE dogs. One month after the initiation of immunosuppressants, CCECAI scores were significantly decreased while NLR values were significantly increased compared to those recorded at the time of diagnosis and, in contrast to a previous study, no significant difference was found between responders and nonresponders [18]. These results might be explained by the high doses of immunosuppressive drugs used here to obtain clinical remission for 2–3 weeks before considering dose reduction [42]. Therefore, NLR seems to have limited clinical utility in predicting treatment response shortly after diagnosis in this population of dogs. Traditionally, anti-inflammatory and immunosuppressive drugs are slowly tapered over weeks, thus reducing their effects on the NLR [43]. It would be, therefore, interesting to monitor the NLR at a longer follow-up.

According to previous studies, a negative correlation between the NLR and the median serum albumin concentration was found [18,19]. It is usually assumed that intestinal inflammation is very severe during PLE, and the serum albumin concentration is commonly decreased [37]. In addition to intestinal inflammation, other factors such as bacterial translocation, local necrosis or loss of lymphocytes in the intestinal lumen may cause an increase in neutrophils [24,40]. In contrast with Becker and colleagues, the NLR here did not differ significantly among the three hypoalbuminemia classes [19]. This result is unexpected; however, it may be explained by the different canine population considered here, the low numerosity of the severe hypoalbuminemia class and the low sensitivity and specificity of the NLR for intestinal inflammation, as highlighted above [15]. In addition, other unknown mechanisms may have played a role, as observed in humans with Crohn’s disease, where a surprising lack of correlation between protein loss and disease activity has been observed [44,45].

Albumin and globulin are negative and positive acute-phase proteins, respectively, and their measurement forms part of most routine biochemistry panels, with AGR expected to decrease in inflammatory and infectious conditions. The evaluation of albumin and globulin alone is influenced by dehydration and fluid retention, whereas the evaluation of their ratios eliminates these effects, reflecting the nutritional and inflammatory status of the body more appropriately [21,22]. Recently, an association between low serum AGR and active disease in IBD patients was observed, as well as between low serum AGR and relapse in pediatric Crohn’s disease patients [22,46]. To the best of the authors’ knowledge, the evaluation of AGR has not been reported in dogs with CE. In this study, the median AGR of dogs with IRE-PLE was significantly lower than that of healthy dogs; moreover, the serum concentrations of AGR significantly decreased with the increase in the CCECAI score. The balance between nutritional and disease effects on the production of negative acute-phase proteins might contribute to hypoalbuminemia and low AGR [47,48]. A recent hypothesis is that the acute-phase response has a stronger effect than the nutritional plane on concentrations of albumin and other negative acute-phase proteins [49]. One month after the initiation of immunosuppressants, AGR was significantly increased, while no difference was found between responders and nonresponders. Although therapy had a positive impact on albumin and AGR, it seems that AGR is not useful in predicting the clinical response in this population of dogs, as assessed by the CCECAI score. Several explanations may be attempted. Firstly, the severity of intestinal inflammation could play a less important role in the clinical response than the immunological response ability of each singular dog. Secondly, although the therapeutic treatment of our study population was very similar, the lack of standardization in terms of the dosage and type of drugs, diet and supplementations used may have influenced the clinical response. Furthermore, since the clinical response may require more than 4 weeks, we cannot exclude different results from monitoring the AGR at a longer follow-up. Finally, the sensitivity and specificity of the ratio for detecting clinical or subclinical disease are not as high as those of positive acute-phase proteins such as CRP [50].

A significant increase in the CRP/ALB with the increase in the CCECAI score was observed before the endoscopic procedure, while a significant decrease in the CRP/ALB and CCECAI score was noted 1 month after the initiation of immunosuppressants. However, the CRP/ALB was not statistically different between responders and nonresponders. Compared with the NLR, the CRP/ALB could be used as an inflammatory biomarker of disease severity and therapeutic efficacy; however, it cannot be used to predict the clinical response in this population of dogs. In the human counterpart, similar results have been recorded [35,51,52], while no information exists in dogs with CE and inflammatory PLE.

The present study had limitations. Firstly, data collection was retrospective. Secondly, it cannot be excluded that new associations would be found including a higher number of dogs. Thirdly, the lack of therapeutic standardization on the levels of inflammatory markers may have played a role. Finally, it cannot be ruled out that different associations would be found monitoring the inflammatory markers at longer follow-up and using different classifications of disease severity.

## 5. Conclusions

Despite the aforementioned limitations, our findings add to previous studies on dogs with chronic enteropathies. The results suggest that AGR and CRP/ALB are potentially more appropriate markers of disease severity and treatment efficacy than NLR in dogs with inflammatory PLE. However, since NLR, AGR and CRP/ALB measurements are influenced by many factors including the use of different laboratory procedures and the different sensitivity and accuracy of hematological and biochemical analyzers, caution is advised in their use and interpretation. Prospective studies are needed to provide more useful information on this subject.

## Figures and Tables

**Table 1:** Selected clinicopathological characteristics of healthy dogs.

Parameter	N	Value
Age (months)	67	72 (24–108)
Sex (female/male)	68	25/43
Weight (kg)	57	21 (12–32)
Neutrophil count (×10^9^ L)	68	5.63 (4.7–6.58)
Lymphocyte count (×10^9^L)	68	2.22 (1.7–3.22)
NLR	68	2.42 (1.83–3.41)
Albumin (g/dL)	61	3.11 (3–3.32)
Globulin (g/dL)	61	3.46 (3.1–3.8)
AGR	61	0.93 (0.82–1.07)

N = number of dogs for which a selected parameter was available. NLR = neutrophil to lymphocyte ratio. AGR = albumin to globulin ratio. Values are expressed as median and interquartile ranges in parentheses.

**Table 2:** Selected clinicopathological characteristics of dogs with IRE-PLE before endoscopic procedure (T0).

Parameter	N	Value
Age (months)	53	84 (68–108)
Sex (female/male)	53	26/27
Weight (kg)	51	17 (6.8–24.7)
CCECAI score	50	9 (8–12)
Disease severity groups		
MI (CCECAI 4–5)		4 (8%)
MO (CCECAI 6–8)		15 (30%)
S (CCECAI 9–11)		13 (26%)
V (CCECAI ≥ 12)		18 (36%)
Neutrophil count (×10^9^ L)	53	9.27 (7.27–12.61)
Lymphocyte count (×10^9^ L)	53	1.63 (1.2–2.06)
NLR	53	5.74 (4.35–8.24)
Albumin (g/dL)	53	1.5 (1.3–1.9)
Hypoalbuminemia classes		
MIA (1.5–2.8 g/dL)		29 (54.7%)
MOA (1.2–1.49 g/dL)		19 (35.9%)
SA (<1.2 g/dL)		5 (9.4%)
Globulin (g/dL)	53	2.1 (1.6–2.4)
AGR	53	0.824 (0.6–1)
CRP (mg/dL)	24	1.39 (0.5–4.5)
CRP/ALB	24	0.95 (0.31–3.43)

N = number of dogs for which a selected parameter was available. NLR = neutrophil to lymphocyte ratio. AGR = albumin to globulin ratio. CRP = C-reactive protein. CRP/ALB = C-reactive protein to albumin ratio. MIA = mild hypoalbuminemia. MI = mild disease. MOA = moderate hypoalbuminemia. MO = moderate disease. S = severe disease. SA = severe hypoalbuminemia. V = very severe disease. Values are expressed as median and interquartile ranges in parentheses unless indicated otherwise by a percentage sign.

**Table 3:** Results of the robust regression studying the relationship between serum concentrations of albumin, AGR and CRP/ALB and the CCECAI score before endoscopic procedure (T0).

	Coefficient	Std. Err.	t	*p* > t	96% CI	
Albumin	−0.0504471	0.0151	−3.34	0.002	−0.0808	−0.0200935
AGR	−0.0252888	0.0118	−2.14	0.037	−0.04901	−0.0015685
CRP/ALB	0.2463005	0.08132	3.03	0.007	0.076669	0.4159323

**Table 4:** The results of the Wilcoxon matched-pairs test used to compare dogs with IRE-PLE before endoscopic procedures (T0) and 1 month after initiation of immunosuppressants (T1).

Parameter	*p* Value
CCECAI	0.000
NLR	0.035
Albumin	0.0001
AGR	0.044
CRP/ALB	0.031

CCECAI = chronic canine enteropathy clinical activity index; NLR = neutrophil to lymphocyte ratio; AGR = albumin to globulin ratio; CRP = C-reactive protein; CRP/ALB = C-reactive protein to albumin ratio.

## Data Availability

The data presented in this study are available upon request from the corresponding author.
